# Papel Diagnóstico do NT-proBNP em Pacientes com Comprometimento por Amiloidose Cardíaca: Uma Metanálise

**DOI:** 10.36660/abc.20210486

**Published:** 2022-05-04

**Authors:** Yingwei Zhang, Hasi Chaolu

**Affiliations:** 1 First Hospital Shanxi Medical University Taiyuan China First Hospital of Shanxi Medical University, Yingze District, Taiyuan – China

**Keywords:** Amiloidose, Diagnóstico, Metanálise em Rede

## Abstract

**Fundamento:**

A amiloidose é definida como um distúrbio caracterizado pela deposição de material proteico amiloide extracelular nos tecidos.

**Objetivos:**

O N-terminal pró-peptídeo natriurético tipo-B (NT-proBNP) é usado para prever a amiloidose cardíaca (AC), mas seu efeito diagnóstico no comprometimento por AC ainda não é claro, especialmente em termos de especificidade e sensibilidade.

**Métodos:**

Foi feita uma busca de literatura nos bancos de dados Pubmed, Embase e a biblioteca Cochrane, e o QUADAS 2 foi utilizado para avaliação da qualidade. O comando Midas no Stata 12.0 foi usado para analisar os indicadores dos sujeitos. O teste Q de Cochran e o I^2^ foram usados como testes de heterogeneidade, e a heterogeneidade significativa foi definida como p <0,05 e/ou I^2^ >50%. A análise de correlação de Spearman foi usada para avaliar o efeito de limiar, e o viés da publicação foi avaliado pelo teste de assimetria. A significância estatística foi definida em p <0,05.

**Resultados:**

Como resultados, 10 conjuntos de dados de 7 estudos foram incluídos para análise, apresentando alta qualidade metodológica e pequenos vieses de confusão. A sensibilidade e a especificidade do NT-proBNP no diagnóstico do comprometimento cardíaco para pacientes com amiloidose foram 0,93 e 0,84, respectivamente. As curvas ROC também sugeriram uma validade diagnóstica alta do NT-proBNP com AUC de 0,95. Um nomograma de Fagan demonstrou que as probabilidades de NT-proBNP positivo e negativo no avanço do comprometimento por AC eram de 90% e 8%, respectivamente. O gráfico de funil de Deek não sugeriu viés significativo de publicação entre os estudos incluídos, e os resultados foram estáveis e confiáveis.

**Conclusões:**

O NT-proBNP desempenha um papel positivo no diagnóstico precoce do comprometimento por AC, com alta sensibilidade e especificidade.

## Introdução

A amiloidose é definida como um distúrbio caracterizado pela deposição de material proteico amiloide extracelular nos tecidos, e ela é patologicamente causada por clivagem, desnaturação ou produção excessiva de proteína anormal.^1 , 2^ O coração é o principal órgão afetado por vários tipos fibrosos de amiloidose.^2^ A amiloidose cardíaca (AC) é uma cardiomiopatia invasiva causada por amiloidose que pode levar à insuficiência cardíaca e distúrbio de condução.^3^ A prevalência do comprometimento de AC na população geral varia entre 5% e 74%, e a ampla diferença sobre variabilidade de pesquisa está associada aos critérios de seleção da população e às estratégias diagnósticas.^4^ As principais causas de comprometimento por AC são proteínas mal dobradas e depósitos de cadeias leves de imunoglobulina amiloide (AL) e proteínas transtirretinas amiloides (ATTR), que podem ser induzidas pela mutação do gene TTR.^5^ A heterogeneidade fenotípica e atrasos no diagnóstico causados por comorbidades contribuem para o prognóstico ruim do comprometimento cardíaco para pacientes com amiloidose.^6^ Muitos casos de comprometimento por AC geralmente são confirmados na tardiamente na evolução da doença quando as opções de tratamento são limitadas.^7^ Portanto, aumentar o entendimento do comprometimento de AC e desenvolver biomarcadores relacionados à amiloidose para um diagnóstico precoce vai efetivamente melhorar o resultado clínico dos pacientes.

O peptídeo natriurético tipo B (BNP) é um tipo de hormônio secretado por miócitos, e podem funcionar na manutenção da homeostase dos fluidos pela ação do sódio, diurese e vasodilatação.^8^ O peptídeo natriurético tipo B N-terminal (NT-proBNP) é clivado para proBNP, que é secretado por cardiomiócitos.^8^ Considera-se que o NT-proBNP seja diretamente regulado por cadeia leve e possa ser usado como biomarcardor para amiloidose AL após análise e validação.^9^ Entretanto, um estudo relevante indicou que o NT-proBNP pode ser um biomarcador sensível, mas não específico, para a avaliação da AC.^10^ Palladini et al. também demonstraram que a gravidade da disfunção cardíaca em pacientes com AC poderia ser avaliada por biomarcadores cardíacos NT-proBNP e troponinas cardíacas (cTn) e que suas avaliações eram altamente sensíveis.^11^ Outras incertezas em relação ao papel do NT-proBNP na previsão do comprometimento por AC tem origem principalmente nas limitações do tamanho da amostra.^12^ Considerando-se as controvérsias de pesquisa acima, espera-se que sejam realizados estudo de porte relevantemente maior para explorar o papel independente e a especificidade diagnóstica do NT-proBNP para prever o comprometimento por AC.

Portanto, conduzimos esta metanálise para obter uma amostra maior, integrando dados de estudos anteriores, e para avaliar o valor diagnóstico do NT-proBNP para comprometimento por AC a partir de vários aspectos, tais como, sensibilidade, especificidade, razões de probabilidade e outros. Este estudo apresenta um marcador diagnóstico para comprometimento cardíaco em pacientes com amiloidose, o qual pode ajudar os pacientes a receber diagnósticos e tratamentos precoces mais precisos.

## Métodos

### Estratégia de coleta da literatura

Foi feita uma busca de literatura nos bancos de dados Pubmed ( https://pubmed.ncbi.nlm.nih.gov/ ), Embase ( https://www.embase.com/ ) e a biblioteca Cochrane ( https://www.cochranelibrary.com/) com data de corte em 28 de janeiro de 2021, e as palavras-chave incluíram: 1) Amiloidose OR amiloidoses; 2) cardiomiopatia OR (comprometimento cardíaco) OR (comprometimento do coração) OR (disfunção miocárdica); 3) NT-proBNP OR (pró-hormônio N-terminal do peptídeo natriurético cerebral) OR (Pró-Peptídeo Natriurético Cerebral N-Terminal). Esses três grupos de palavras-chave foram combinados com “AND”. Além disso, palavras sobre o tema e palavras livres foram combinadas na pesquisa, e as estratégias de coleta variaram de acordo com as características de três bancos de dados. O processo de coleta detalhado e os resultados relacionados estão apresentados na Tabela suplementar 1-3 . Ademais, a versão impressa da literatura foi coletada manualmente, e as referências dos artigos incluídos e resenhas relevantes também foram triadas de acordo com o critério de inclusão.

### Seleção das publicações

Os critérios de inclusão foram os seguintes: 1) sujeitos com amiloidose AL ou amiloidose relacionada ao TTR; 2) sujeitos com disfunção do ventrículo direito/esquerdo, insuficiência cardíaca e outras disfunções cardíacas diagnosticadas por imagens por ressonância magnética cardíaca ou biópsia; 3) apresentação de resultados diagnósticos de injúria cardíaca causada pelo NT-proBNP incluindo verdadeiro positivo (VP), falso positivo (FP), verdadeiro negativo (VN) e falso negativo (FN), ou que podem ser extrapolados de acordo com dados da literatura. Publicações que não são artigos originais, tais como resenhas, cartas, comentários e outros, foram excluídas deste estudo.

### Aquisição de dados e avaliação de qualidade

Dois pesquisadores registraram os dados independentemente de acordo com um formulário padronizado elaborado com antecedência. As informações coletadas incluíram o nome do primeiro autor, ano de publicação, área de estudo, tamanho da amostra, idade e sexo dos sujeitos, dados de VP, FP, VN e FN, e critérios para lesões cardíacas. Após a extração dos dados, foi conduzida uma discussão para resolver inconsistências. O QUADAS 2 foi aplicado para avaliar a qualidade dos métodos de pesquisa usados em cada estudo incluído.^13^

### Análise estatística

O comando Midas (modelo de efeito misto bivariado) do Stata 12.0 versão 12 SE (Stata Corporation, TX, EUA) foi aplicado para a análise estatística de índices de sujeitos, incluindo a curva sumária de características de operação do receptor (SROC), sensibilidade, especificidade, razões de probabilidade positiva (RPP), razões de probabilidade negativa (RPN), razão de chance diagnóstica (RCD), e intervalos de confiança (IC) de 95%. O valor da RCD variou de 0 a infinito, e o valor mais alto indicou a capacidade discriminatória maior dos métodos diagnósticos.^14^ A curva SROC foi estabelecida com base em sensibilidade e especificidade, e quanto mais próxima a área sob a curva (AUC) estiver de 1, mais alta será a validade diagnóstica.^15^ Os testes Q de Cochran e I^2^ foram usados para avaliar a heterogeneidade,^16^ e p > 0,05 e/ou I^2^ > 50% indicava heterogeneidade significativa entre os estudos. A análise de correlação de Spearman foi usada para avaliar o efeito de limiar, e p <0,05 indicava um efeito de limiar significativo.^17^ O gráfico de funil de Deek foi usado para avaliar se havia viés de publicação significativo entre os estudos,^18^ enquanto o nomograma de Fagan foi usado para avaliar a utilidade clínica do NT-proBNP.^19^ Foi realizada a análise de sensibilidade usando um modelo gráfico para avaliar se o mesmo tinha algum possível erro de especificação, bondade do ajuste, para identificar pontos de dados anormais e possivelmente influentes.^20^

## Resultados

### Triagem da literatura

**Figura 1:**
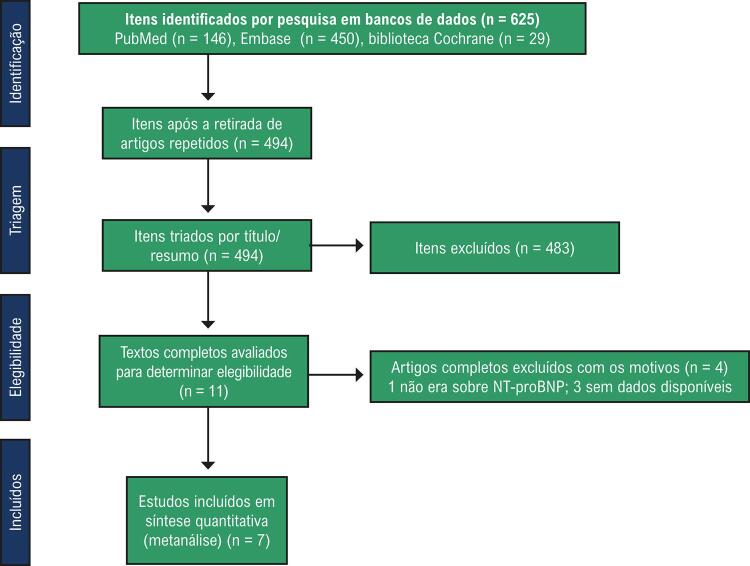
– Os processos e resultados da busca de literatura.

Os processos e resultados da coleta de literatura são apresentados na Figura 1 . Foram obtidos 450, 146 e 29 artigos dos bancos de dados de Embase, PubMed e biblioteca Cochrane, respectivamente. Um total de 494 artigos passaram pela triagem após a eliminação de duplicatas. Entre eles, 483 artigos foram retirados após a leitura dos títulos e resumos. Em seguida, após a leitura integral dos artigos, 4 outros artigos também foram eliminados. Além disso, a busca manual não conseguiu identificar publicações que atenderam aos requisitos. Por fim, 7 artigos^12 , 21 - 26^ foram incluídos nesta análise.

### Características dos artigos incluídos

Um total de 7 artigos foi incorporado a este estudo. Entre eles, o estudo de Nicol et al.^25^ continha dois conjuntos de dados, o estudo de Palladini et al.^26^ continha três conjuntos de dados, e os outros cinco estudo continham um conjunto de dados cada um. Portanto, foram incluídos 10 conjuntos de dados no total para análises posteriores. Esses sete estudos, publicados entre 2003 e 2020, envolvendo 810 sujeitos no total (incluindo 490 pacientes com comprometimento por AC e 320 controles), e foram conduzidos na Holanda, na Alemanha, na França, na Itália e em outros países. Além disso, todos os níveis de NT-proBNP foram detectados por imunoensaio nos estudos incluídos. Entre eles, 4 estudos focaram em amiloidose AL, 2 estudos incluíram amiloidose TTR, e os demais estudos incorporaram amiloidose AL e TTR. Entretanto, a amiloidose foi confirmada por biópsia em 6 estudos, exceto no estudo de Damy et al.,^21^ que não informaram a estratégia de diagnóstico. As características desses 7 estudos, incluindo os critérios de comprometimento cardíaco e limiares diagnósticos, foram sintetizadas na Tabela 1 . Entre os 7 estudos incluídos, 5^12 , 21 - 23 , 25^ geraram análise diferencial sobre a idade dos sujeitos, e 4 estudos^12 , 21 - 23^ compararam a diferença de sexo entre casos e controles. Nesses estudos, Damy et al.^21^ encontraram diferenças significativas associadas ao sexo e à idade dos sujeitos do estudo (p <0,05); as amostras incluídas por Klaassen et al. foram significativamente diferentes em idade (p < 0,05); e outras comparações com p ≥ 0,05 foram consideradas como equivalentes em relação a idade e/ou sexo. Outros três estudos de Nicol^25^ e Palladini et al.^24 , 26^ não compararam diferenças de idade e/ou sexo. Em seguida, foi utilizado o QUADAS 2 para fazer a avaliação de qualidade das publicações, e os resultados demonstraram um risco baixo de viés e alta qualidade da metodologia dos estudos envolvidos ( Figura suplementar 1 ).

**Tabela 1:** Características dos 7 estudos incluídos nesta metanálise

Estudo	Área	Comprovação da amiloidose	Tipo de amiloidose	Critério de comprometimento cardíaco	N	Caso/Controle			Corte, pg/ml	VP	FP	FN	VN

						n	Idade, anos	Sexo masculino, n (%)					
Cappelli, F^12^ 2014	Itália	Biópsia	AL	DVD	76	23/53	70,7±9,2/ 68,9±10,1	9 (39,1)/ 24 (45,3)	≥2977	20	8	3	45
Damy, T^21^ 2013	França	NR	TTR	DVE	36	26/10	65(56-74)/ 40 (33-56) *	20 (76,9)/ 3 (30,0) *	≥82	24	1	2	9
Klaassen, SHC^22^ 2017	Holanda	Biópsia	TTR	Anormalidades da parede miocárdica estrutural e/ou distúrbio de condução	77	39/38	59,3±10,9/ 46,1±13,0 *	25 (64,1)/ 18 (47,4)	≥125	36	13	3	25
Lehrke, S^23^ 2009	Alemanha	Biópsia	AL ou TTR	Biópsia cardíaca positiva e/ou HVE	34	25/9	55,5±11,0/ 59,8±7,8	10 (40,0)/ 6 (66,7)	≥1736,5	23	3	2	6
Nicol, M^25^ 2020	França	Biópsia	AL	RMC e biópsia endomiocárdica	114	82/32	66 (58-73)/ 68 (60-76)	NR	≥850	75	8	7	24
					73	48/25	NR	NR	≥850	44	1	4	24
Palladini, G^24^ 2003	Itália	Biópsia	AL	Sintomas clínicos de insuficiência cardíaca, HVE	152	90/62	61 (34-78) #	NR	≥152	84	6	6	56
Palladini, G^26^ 2012	Itália	Biópsia	AL	Espessura da parede do ventrículo esquerdo > 12 mm	109	62/47	62 (29-83) #	63 (58) #	≥332	62	5	0	42
					77	54/23	64 (35-85) #	34 (44) #	≥543	50	2	4	21
					62	41/21	65 (38-82) #	33 (53) #	≥2642	38	6	3	15

AL: cadeia leve de amiloide; TTR: relacionada à transtirretina hereditária; RMC: ressonância magnética cardíaca; DVD: disfunção do ventrículo direito; DVE: disfunção do ventrículo esquerdo; HVE: hipertrofia do ventrículo esquerdo; NR: não relatado; VP: verdadeiro positivo; FP: falso positivo; FN: falso negativo; VN: verdadeiro negativo. #, dados da amostra total. As significâncias estatísticas de todos os estudos, com exceção do de Palladini et al.^24,26^, foram definidas como p <0,05, e * indica a diferença estatística.

### Valor diagnóstico do NT-proBNP

**Figura 2:**
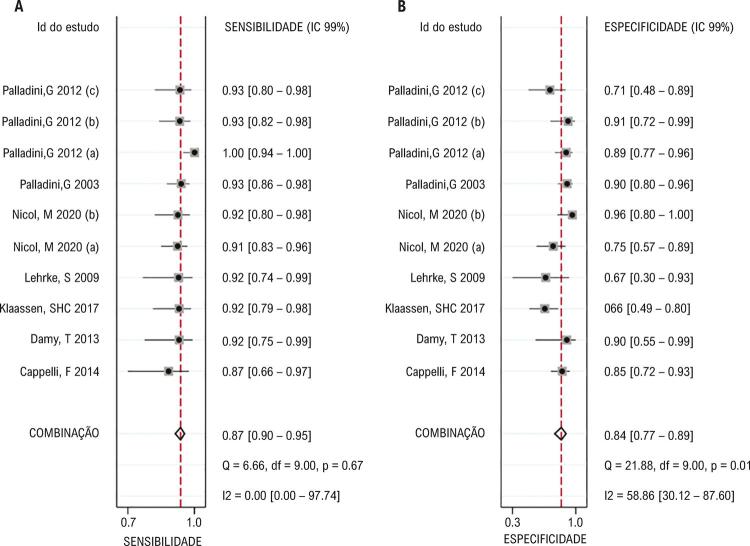
– Gráficos de floresta de estimativas de sensibilidade (A) e especificidade (B) no diagnóstico do NT-proBNP para lesão cardíaca de 10 conjuntos de dados.

**Figura 3:**
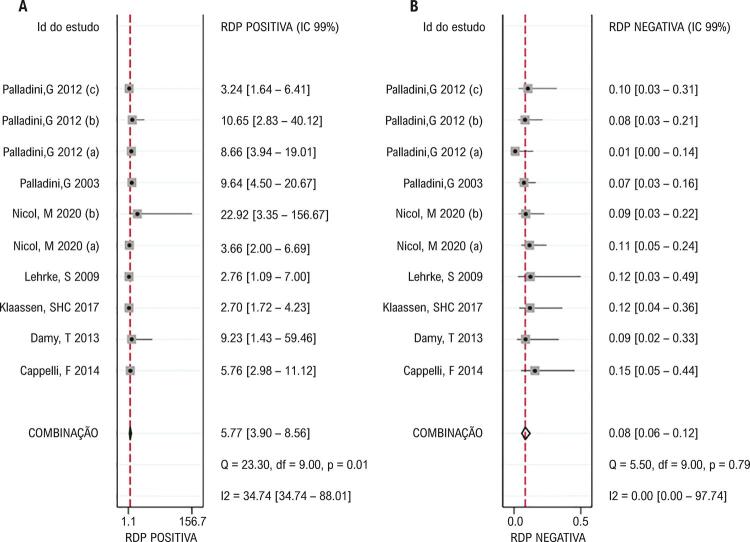
– Gráficos de floresta de estimativas de RPP (A) e RPN (B) no diagnóstico do NT-proBNP para lesão cardíaca de 10 conjuntos de dados.

**Figura 4:**
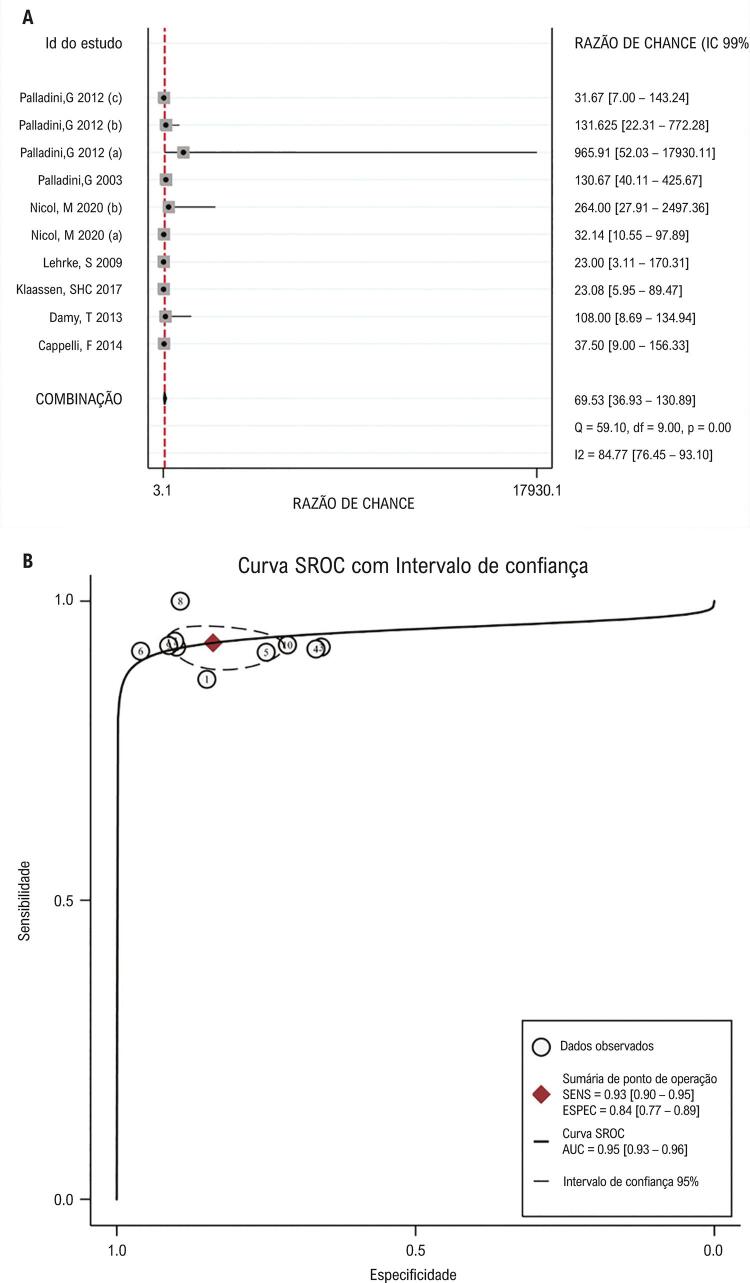
– Validade diagnóstica do NT-proBNP para lesão cardíaca. A. Gráficos de floresta de estimativas de RCD de 10 conjuntos de dados. B. As curvas SROC mostram a validade diagnóstica do NT-proBNP na lesão cardíaca com AUC de 0,95.

Um total de 7 artigos (10 conjuntos de dados de população) relataram os resultados dos níveis de NT-proBNP no diagnóstico das lesões cardíacas em pacientes com amiloidose, e a análise de correlação de Spearman sugeriu que *p* = 1,00 é o resultado que indicou que não havia efeito de limiar significativo. Em seguida, foi estabelecido um modelo de efeito misto bivariado para investigar o valor diagnóstico do NT-proBNP na lesão cardíaca com base em indicadores diferentes, e os testes de Cochran e I^2^ foram aplicados para analisar a heterogeneidade entre estudos. Os resultados ( Figura 2 ) demonstraram que a sensibilidade e a especificidade estimadas eram 0,93 e 0,84, respectivamente. Não houve heterogeneidade significativa em sensibilidade (p = 0,67, I^2^ = 0,0%), mas houve uma heterogeneidade significativa em especificidade (p = 0,01, I^2^ = 58,86%) entre os estudos. Na Figura 3 , o valor combinado da RPP era 5,77 com uma heterogeneidade significativa entre estudos (p = 0,01, I^2^ = 34,74%), enquanto o valor para RPN era 0,80 sem heterogeneidade significativa (p = 0,79, I^2^ = 0,0%). A Figura 4A mostra que esses conjuntos de dados eram significativamente heterogêneos na RCD (p <0,01, I^2^= 84,77%), com uma estimativa combinada de 69,53 A AUC da SROC era 0,95 e esses estudos não foram significativamente distribuídos em uma forma curvilínea ( Figura 4B ), sugerindo uma grande validade diagnóstica do NT-proBNP na lesão cardíaca.

### Utilidade clínica do NT-proBNP

**Figura 5:**
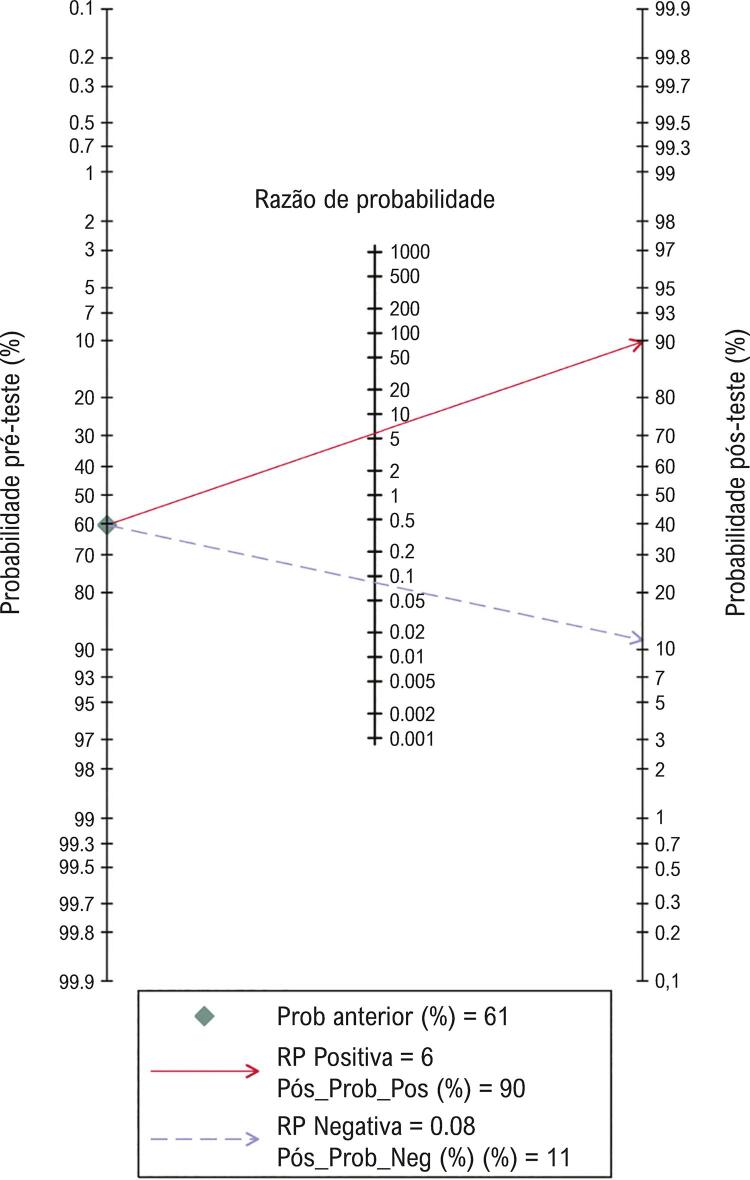
– Utilidade clínica do NT-proBNP. O gráfico do nomograma de Fagan mostra a probabilidade pré-teste, a RPP, a RPN e a probabilidade pós-teste do NT-proBNP para o diagnóstico da injúria cardíaca.

Em seguida, foi realizado um nomograma de Fagan para avaliar a utilidade clínica do NT-proBNP, conforme ilustrado na Figura 5 , e o gráfico do nomograma de Fagan apresentou a probabilidade pré-teste, a RPP, a RPN e a probabilidade pós-teste do NT-proBNP no diagnóstico da injúria cardíaca. Os resultados sugeriram que a probabilidade pré-teste com lesão cardíaca era de 60,5%, e a probabilidade pós-teste era 90% e 8% para pacientes com resultado positivo e negativo, respectivamente. Isso significa que, após o diagnóstico do NT-proBNP, a probabilidade de desenvolvimento de lesão cardíaca nas populações com NT-proBNP positivo era de 90%, enquanto a possibilidade em populações com NT-proBNP negativo era de apenas 8%.

### Análise de sensibilidade e teste de viés da publicação

**Figura 6:**
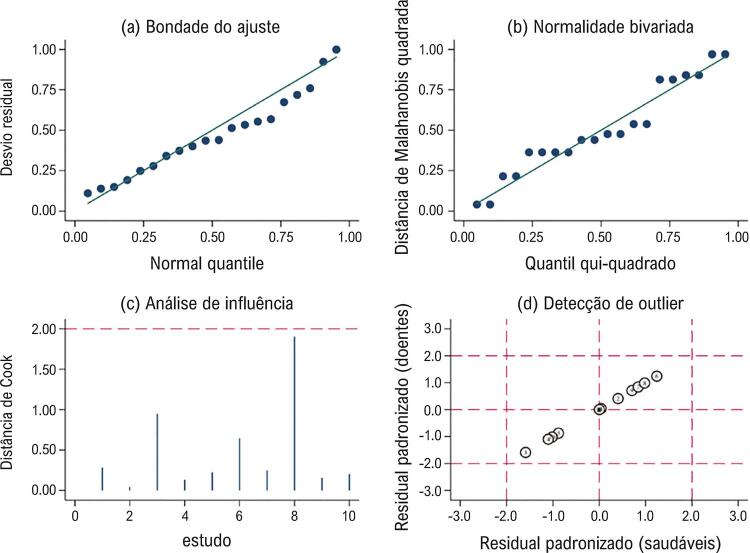
– Análise de sensibilidade em um modelo gráfico. A-B mostram a bondade do ajuste e normalidade bivariada do modelo. C. Análise de influência do estudo independente nos resultados combinados. D. Detecção de outliers do estudo independente.

**Figura 7:**
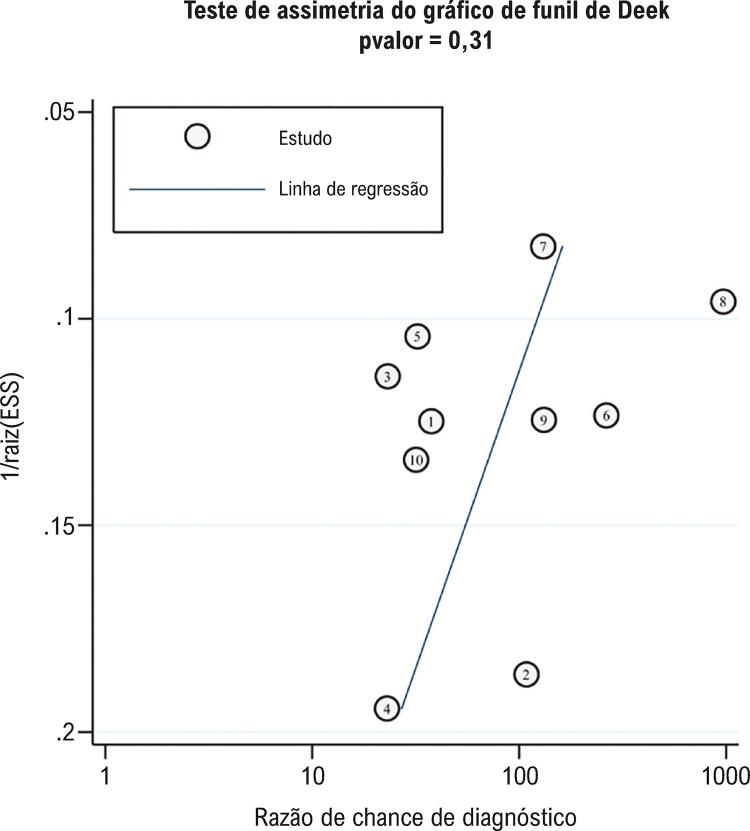
– Teste de viés de publicação. O gráfico de funil de Deek mostra o viés da publicação no teste de assimetria

Em seguida, o modelo gráfico foi realizado para análise de sensibilidade. Os resultados sugeriram uma grande bondade do ajuste de base residual do modelo ( Figura 6A ), que basicamente se conformava à premissa de normalidade bivariada ( Figura 6B ). Também se identificou que cada estudo independente não tinha efeito significativo nos resultados combinados do modelo, e nenhum outlier foi identificado ( Figura 6C-D ). Por último, foi criado um gráfico de funil de Deek para testar o viés da publicação, e os resultados na Figura 7 sugeriram que não havia viés de publicação, com um p = 0,31 no teste de assimetria. Esses achados propõem resultados combinados estáveis e confiáveis nesta metanálise.

## Discussão

O diagnóstico de comprometimento cardíaco em pacientes com amiloidose geralmente é atrasado pela diversidade de manifestações clínicas, resultando, portanto, em um prognóstico ruim.^5^ Relata-se que uma vez que a amiloidose AL apresenta sintomas de insuficiência cardíaca congestiva, pacientes não tratados têm uma sobrevida mediana de menos de 6 meses.^2^ Portanto, é essencial desenvolver um biomarcador relacionado à AC para melhorar a eficiência do diagnóstico precoce. O NT-proBNP tem sido usado como um biomarcador potencial para se avaliar a gravidade do comprometimento cardíaco na amiloidose AL,^27^ mas o papel independente do comprometimento por AC e sua especificidade diagnóstica não foram totalmente investigados. Dessa forma, realizamos esta metanálise com base em 7 artigos, e avaliamos a influência do NT-proBNP no diagnóstico do comprometimento por AC. Nossos resultados sugerem que o NT-proBNP tem valores diagnósticos significativos para lesão cardíaca em pacientes com amiloidose, com uma sensibilidade de 0,93, uma especificidade de 0,84, uma RPP de 5,77, uma RPN de 0,08 e uma RCD de 69,53. A AUC da curva SROC também é próxima a 1 (0,95), demonstrando, portanto, uma grande validade diagnóstica do NT-proBNP.

Relata-se que a destruição local de cardiomiócitos leva a níveis elevados de NT-proBNP, e o aumento do nível de NT-proBNP poderia ser considerado um preditor de comprometimento cardíaco antes do surgimento da insuficiência cardíaca.^7^ Banypersad et al. também identificaram uma correlação entre doença cardíaca e NT-proBNP em 100 pacientes com amiloidose AL detectada por imagens por ressonância magnética nuclear.^28^ Além disso, a mortalidade após 1 ano de 125 pacientes com amiloidose AL pode ser prevista pela análise de estratificação de risco de NT-proBNP e cTn.^29^ Em relação a um mecanismo de regulação potencial da expressão do NT-proBNP no amiloide em cardiomiócitos, Shi et al. propuseram que proteínas de cadeia leve isoladas de tecidos com cardiomiopatia amiloide podem induzir o sinal de proteínas quinases ativadas por mitógenos p38 (MAPK), contribuindo, dessa forma, para o stress oxidativo e a morte de cardiomiócitos.^30^ Além disso, para a amiloidose AL, o sinal MAPK poderia mediar a transcrição de BNP, e sua interação pode suportar o efeito tóxico de proteínas de cadeia leve.^31^ Em combinação com os achados acima, especula-se que a expressão do NT-proBNP poderia ser diretamente regulada pela via de transdução de sinal MAPK induzida por proteínas de cadeia leve em cardiomiócitos, e o aumento do nível de expressão do NT-proBNP pode prever o ataque da insuficiência cardíaca.

Há uma variedade de estudos com foco na influência do NT-proBNP no comprometimento cardíaco incluindo insuficiência cardíaca, cardiomiopatia e infarto do miocárdio. Uma metanálise relacionada relatou que a sensibilidade e a especificidade do nível de NT-proBNP na diferenciação da efusão associada a insuficiência cardíaca era de 94%, com uma RPP de 15,2 e uma RPN de 0,06.^32^ O aumento do nível de NT-proBNP também demonstra uma forte capacidade de prever o prognóstico de cardiomiopatia.^33^ Além disso, pela comparação ao índice de risco cardíaco revisado, o biomarcardor NT-proBNP de alta sensibilidade pode melhorar a previsão do infarto do miocárdio após uma grande cirurgia não cardíaca.^34^ Esses achados corroboram nossas conclusões, mas Januzzzi et al. propuseram ainda que o nível de NT-proBNP estava relacionado à gravidade dos sintomas de insuficiência cardíaca, e a sensibilidade e a especificidade da insuficiência cardíaca variaram entre as várias faixas etárias.^35^ Neste estudo, não se observou uma relação direta entre idade e os níveis de NT-proBNP, e não foi possível confirmar a importância da idade no comprometimento por AC diagnosticado por NT-proBNP. Além disso, estudos também identificaram que sujeitos do sexo feminino têm níveis mais altos de NT-proBNP do que sujeitos do sexo masculino de idades equivalentes.^36^ Portanto, será realizada análise estratificada posteriormente para explorar as diferenças dos marcadores NT-proBNP com base em desempenho analítico, para proporcionar informações diagnósticas mais precisas para pacientes com comprometimento por AC em estratificações clínicas diferentes.

As vantagens deste estudo incluem o fato de que o estudo incorporado tinha metodologia altamente qualificada, e o viés de confusão era pequeno. Além disso, não houve viés de publicação significativo neste estudo, e a análise de influência também sugeria que os resultados combinados não foram afetados por cada estudo independente. Mais importante, os resultados combinados de todos os indicadores foram relativamente consistentes, sugerindo que o NT-proBNP tinha um alto valor de aplicação no diagnóstico de lesão cardíaca em pacientes com amiloidose, e os resultados foram estáveis e confiáveis. Embora nossos resultados sugiram alta sensibilidade e especificidade do NT-proBNP no diagnóstico do comprometimento por AC, a heterogeneidade significativa em especificidade, RPP e RCD entre os estudos incluídos foi uma das limitações. No entanto, também havia diferenças nos critérios diagnósticos, tipos de amiloidose e critérios para se determinar lesões cardíacas entre os sujeitos. Entretanto, devido ao tamanho limitado da amostra de estudos incluídos, é difícil explorar a fonte de heterogeneidade por métodos quantitativos, tais como a metarregressão. Segundo todos os estudos incluídos foram realizados em populações europeias, com uma generalização ruim dos resultados. Ainda é necessário realizar estudos de alta qualidade na Ásia, na África e em outras regiões para validar o desempenho dos resultados.

## Conclusão

Para concluir, este estudo sugere que o NT-proBNP desempenha um papel positivo no diagnóstico precoce do comprometimento cardíaco em pacientes com amiloidose. Estudos em larga escala em outras regiões e raças são necessários para verificar a extrapolação dos resultados.

## * Material suplementar

Para visualizar a figura suplementar, por favor, clique aqui



Para visualizar as tabelas suplementares, por favor, clique aqui.


