# Supplementary material for: Papel Diagnóstico do NT-proBNP em Pacientes com Comprometimento por Amiloidose Cardíaca: Uma Metanálise
Source: Arq Bras Cardiol. 2022 May 4;119(2):212–22. [Article in Portuguese] doi: 10.36660/abc.20210486 (PMC9363055; doi:10.36660/abc.20210486)

Supplemental Figure 1 Quality assessment of included studies.

Quality assessment including risk of bias and concerns regarding applicability of included 7 studies were evaluated. The X-axis indicates proportion of studies with low (blue), high (orange) or unclear (blue), and the Y-axis indicates the domains of studies including flow and timing, reference standard, index test and patients selection.

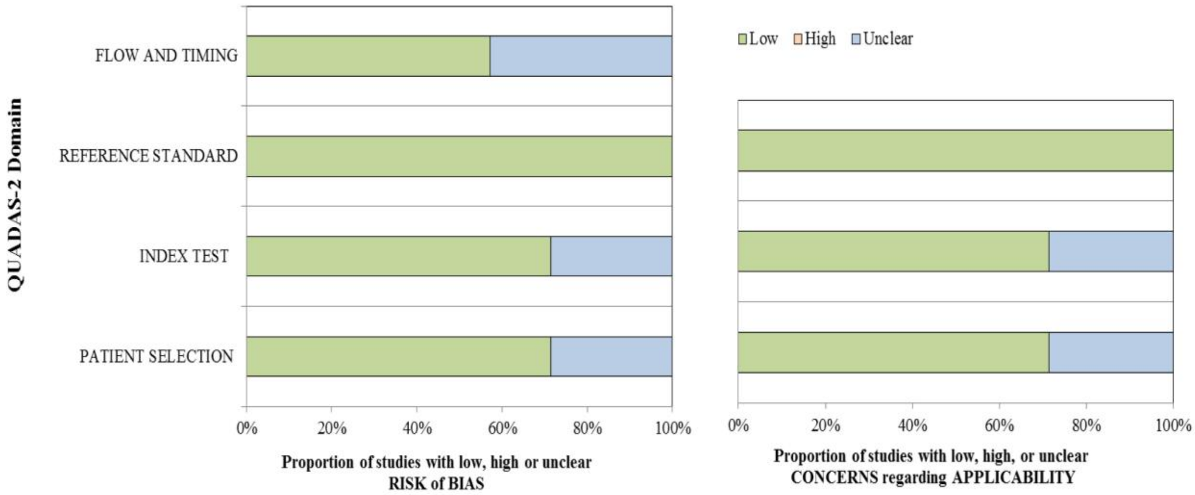

Supplement: Supplementary file 1 [file 2021-0486-supplementary-figure-1.pdf]
