# Supplementary material for: Papel Diagnóstico do NT-proBNP em Pacientes com Comprometimento por Amiloidose Cardíaca: Uma Metanálise
Source: Arq Bras Cardiol. 2022 May 4;119(2):212–22. [Article in Portuguese] doi: 10.36660/abc.20210486 (PMC9363055; doi:10.36660/abc.20210486)
Supplement: Supplementary file 2 [file 2021-0486-supplementary-tables.pdf]

Supplemental Table 1 Retrieval process and results in PubMed

Search	Query	Items found
#1	"amyloidosis"[MeSH Terms] OR "amyloidosis"[All Fields] OR "amyloidoses"[All Fields]	32530
#2	"cardiomyopathie"[All Fields] OR "cardiomyopathies"[MeSH Terms] OR "cardiomyopathies"[All Fields] OR "cardiomyopathy"[All Fields] OR (("cardiacs"[All Fields] OR "cardiac"[All Fields] OR ("heart"[MeSH Terms] OR "heart"[All Fields] OR "hearts"[All Fields] OR "heart s"[All Fields])) AND ("involve"[All Fields] OR "involved"[All Fields] OR "involvement"[All Fields] OR "involvements"[All Fields] OR "involves"[All Fields] OR "involving"[All Fields] OR "involvment"[All Fields])) OR (("myocardially"[All Fields] OR "myocardium"[MeSH Terms] OR "myocardium"[All Fields] OR "myocardial"[All Fields]) AND ("dysfunctional"[All Fields] OR "dysfunctionals"[All Fields] OR "dysfunctioning"[All Fields] OR "dysfunctions"[All Fields] OR "dysfunction"[All Fields]))	281928
#3	"pro brain natriuretic peptide 1 76"[Supplementary Concept] OR "pro brain natriuretic peptide 1 76"[All Fields] OR "NT-probnp"[All Fields] OR ("N-terminal"[All Fields] AND ("prohormonal"[All Fields] OR "prohormone"[All Fields] OR "prohormones"[All Fields]) AND ("natriuretic peptide, brain"[MeSH Terms] OR ("natriuretic"[All Fields] AND "peptide"[All Fields] AND "brain"[All Fields]) OR "brain natriuretic peptide"[All Fields] OR ("brain"[All Fields] AND "natriuretic"[All Fields] AND "peptide"[All Fields]))) OR ("n terminal"[All Fields] AND "Pro-B-Type"[All Fields] AND ("natriuretic peptides"[MeSH Terms] OR ("natriuretic"[All Fields] AND "peptides"[All Fields]) OR "natriuretic peptides"[All Fields] OR ("natriuretic"[All Fields] AND "peptide"[All Fields]) OR "natriuretic peptide"[All Fields]))	9818
#4	#1 AND #2 AND #3	146

The retrieval time was due on 20210128.

Supplemental Table 2 Retrieval process and results in Embase

Search	Query	Items found
#1	('amyloidosis'/exp OR amyloidosis)	46533
#2	NT-proBNP	14943
#3	(('n terminal' AND ('prohormone'/exp OR prohormone) AND of AND ('brain'/exp OR brain) AND natriuretic AND ('peptide'/exp OR peptide)) OR ('n terminal' AND 'pro b type' AND natriuretic AND ('peptide'/exp OR peptide)))	4125
#4	#2 OR #3	16398
#5	('cardiomyopathy'/exp OR cardiomyopathy OR 'cardiac involvement' OR 'heart involvement' OR 'myocardial dysfunction'/exp OR 'myocardial dysfunction')	163869
#6	#1 AND #4 AND #5	450

The retrieval time was due on 20210128.

Supplemental Table 3 Retrieval process and results in Cochrane library

Search	Query	Items found
#1	(amyloidosis OR amyloidoses):ti,ab,kw (Word variations have been searched)	575
#2	MeSH descriptor: [Cardiomyopathies] explode all trees	1991
#3	(Cardiomyopathies OR (cardiac involvement) OR (heart involvement) OR (myocardial dysfunction)):ti,ab,kw (Word variations have been searched)	16147
#4	#2 OR #3	16699
#5	((NT-proBNP) OR (N-terminal prohormone of brain natriuretic peptide) OR (N-Terminal Pro-B-Type Natriuretic Peptide)):ti,ab,kw (Word variations have been searched)	2445
#6	#1 AND #4 AND #5	29
#7	#6 in Trials	29

The retrieval time was due on 20210128.
