# Supplementary material for: Papel Diagnóstico do NT-proBNP em Pacientes com Comprometimento por Amiloidose Cardíaca: Uma Metanálise
Source: Arq Bras Cardiol. 2022 May 4;119(2):212–22. [Article in Portuguese] doi: 10.36660/abc.20210486 (PMC9363055; doi:10.36660/abc.20210486)
Supplement: Translation

# Translation: Diagnostic Role of NT-proBNP in Patients with Cardiac Amyloidosis Involvement: A Meta-Analysis

**Authors:** Yingwei Zhang, Hasi Chaolu

**Affiliations:** 1 China First Hospital of Shanxi Medical University, Yingze District, Taiyuan – China

**Keywords:** Amyloidosis, Diagnosis, Network Meta-Analysis

## Abstract

**Background:**

Amyloidosis is defined as a disorder characterized by the deposition of extracellular protein material of amyloid in tissues.

**Objectives:**

N-terminal pro-B-type natriuretic peptide (NT-proBNP) is used to predict the cardiac amyloidosis (CA), but its diagnostic effect on CA involvement remains unclear, especially in terms of specificity and sensitivity.

**Methods:**

A search for literature was conducted in the Pubmed, Embase, and Cochrane library databases, and QUADAS 2 was used for quality assessment. Midas command in Stata 12.0 was used to analyze the subject indicators. Cochran’s Q and I^2^were to test for heterogeneity, and the significant heterogeneity was set at p < 0.05 and/or I^2^> 50%. Spearman correlation analysis was used to evaluate the threshold effect, and the publication bias was assessed using the asymmetry test. The statistical significance was set at p < 0.05.

**Results:**

As results, 10 sets of data from 7 studies were included for analysis, showing high methodological quality and minimal confounding bias. The sensitivity and specificity of NT-proBNP in the diagnosis of cardiac involvement for patients with amyloidosis were 0.93 and 0.84, respectively. ROC curves also suggested a high diagnostic validity of NT-proBNP with an AUC of 0.95. A Fagan’s nomogram plot showed probabilities for NT-proBNP positive and negative in developing CA involvement were 90% and 8%, respectively. The Deek’s funnel plot suggested no significant publication bias across included studies, and the results were stable and reliable.

**Conclusions:**

NT-proBNP plays the positive role in the early diagnosis of CA involvement with high sensitivity and specificity.

## Introduction

Amyloidosis is defined as a disorder characterized by the deposition of extracellular protein material of amyloid in tissues, and it is pathologically caused from cleavage, denaturation or excessive production of abnormal protein.^1 , 2^The heart is the main affected organ of different fibrous types of amyloidosis.^2^Cardiac amyloidosis (CA) is an invasive cardiomyopathy caused by amyloidosis, and may give rise to heart failure and conductive disease.^3^The prevalence of CA involvement in the general population ranges from 5%-74%, and the wide differences in research variability are associated with population selection criteria and diagnostic strategies.^4^Protein misfolds and deposits of amyloid immunoglobulin light chain protein (AL) and amyloid transthyretin (TTR) proteins, which may be induced by the mutation of TTR gene, are the main causes of CA involvement.^5^Phenotypic heterogeneity and delays in diagnosis caused by comorbidities contribute to the poor prognosis of cardiac involvement for patients with amyloidosis.^6^Many cases of CA involvement are usually confirmed in the disease course of late with limited treatment options.^7^Therefore, increasing the understanding of CA involvement and developing amyloidosis-related biomarkers for early diagnosis will effectively improve the clinical outcome of patients.

B-type natriuretic peptide (BNP) is a type of hormone secreted by myocyte cells, and may function in maintaining fluid homeostasis through the action of sodium, diuresis, and vasodilation.^8^N-terminal pro-BNP (NT-proBNP) is cleaved to proBNP, which is secreted by cardiomyocytes.^8^NT-proBNP is considered to be directly regulated by light chain and can be used as a biomarker for AL amyloidosis after analysis and validation.^9^However, one relevant study pointed out that NT-proBNP may be a sensitive but non-specific biomarker for the assessment of CA.^10^Palladimi et al. also illustrated that the severity of cardiac dysfunction in patients with CA could be assessed by NT-proBNP cardiac biomarkers and cardiac troponins (cTn), and their evaluations were highly sensitive.^11^Other incertitudes regarding the role of NT-proBNP in predicting CA involvement stem mainly from the limitations of sample size.^12^In light of the above research controversies, studies with a relevantly larger size are warranted in order to explore the independent role and diagnostic specificity of NT-proBNP to predict the CA involvement.

Therefore, this meta-analysis was conducted to obtain a larger sample size by integrating data from previous studies and to evaluate the diagnostic value of NT-proBNP for CA involvement from various aspects, including sensitivity, specificity, likelihood ratios, among others. Our study provides a diagnostic marker for cardiac involvement in patients with amyloidosis, which may help patients to receive more accurate early diagnosis and treatment.

## Methods

### Literature retrieval strategy

A search for literature was conducted in the Pubmed ( https://pubmed.ncbi.nlm.nih.gov/ ), Embase ( https://www.embase.com/ ) and Cochrane library ( https://www.cochranelibrary.com/ ) databases with the deadline date of January 28, 2021, and the key words included: 1) Amyloidosis OR amyloidoses; 2) cardiomyopathy OR (cardiac involvement) OR (heart involvement) OR (myocardial dysfunction); 3) NT-proBNP OR (N-terminal prohormone of brain natriuretic peptide) OR (N-Terminal Pro-B-Type Natriuretic Peptide). These three groups of key words were combined with “AND”. Moreover, subject words and free words were combined in the search, and the retrieval strategies varied according to the characteristics of three databases. The detailed retrieval process and related results were shown in Supplemental Table 1-3 . Furthermore, the paper version of literatures was manually retrieved, and the references of the included literatures and relevant reviews were also screened according to the inclusion criteria.

### Publication selection

The inclusion criteria were as follows: 1) subjects with AL amyloidosis or TTR-related amyloidosis; 2) subjects with left/right ventricular dysfunction, heart failure, and other cardiac dysfunction diagnosed by cardiac magnetic resonance imaging or biopsy; 3) provided diagnostic results of NT-proBNP-caused cardiac injury including true positive (TP), false positive (FP), true negative (TN) and false negative (FN), or can be extrapolated according to data from the literature. Non-treatise literatures, such as reviews, letters, comments, among others, were excluded from this study.

### Data acquisition and quality evaluation

The two investigators independently logged the data according to a standardized form designed in advance. The acquired information included the name of the first author, publication year, study area, sample size; age and sex of subjects; data of TP, FP, TN and FN; and criteria for heart damage. After data extraction, discussion was conducted to solve the inconsistency. QUADAS 2 was applied to assess the quality of research methods used in each included study.^13^

### Statistical analysis

Midas command (bivariate mixed-effect model) in Stata12.0 version 12 SE (Stata Corporation, TX, USA) was applied for statistical analysis on indexes of subjects including summary receiver operating characteristic (SROC) curve, sensitivity, specificity, positive likelihood ratios (PLR), negative likelihood ratios (NLR), diagnostic odds ratio (DOR), and 95% confidence intervals (CI). The value of DOR ranged from 0 to infinite, and the larger value indicated the greater discriminatory ability of diagnostic methods.^14^The SROC curve was established based on sensitivity and specificity, and the closer the area under the curve (AUC) to 1, the higher the diagnostic validity.^15^Cochran’s Q and I^2^tests were used to evaluate the heterogeneity,^16^and p > 0.05 and/or I^2^> 50% indicated significant heterogeneity between studies. Spearman correlation analysis was used to assess the threshold effect, and p < 0.05 indicated a significant threshold effect.^17^Deek’s funnel plot was used to evaluate whether there was significant publication bias between studies,^18^while Fagan’s nomogram was used to evaluate the clinical utility of NT-proBNP.^19^Sensitivity analysis was performed using a graph model to evaluate whether or not it contained possible misspecifications, goodness of fit, identify outlying, and possibly influential data points.^20^

## Results

### Literature screening

The process and results of literature retrieval were shown in Figure 1 . We obtained 450, 146, and 29 articles from Embase, PubMed and Cochrane library databases, respectively. A total of 494 articles were screened after having eliminated duplicates. Among these, 483 articles were removed after reading the titles and abstracts. After reading the full paper, 4 articles were further eliminated. Furthermore, the manual search failed to screen the publications that met the requirements. Finally, 7 articles^12 , 21 - 26^were included in this analysis.

**Figure 1:** – The process and results of literature retrieval.

### Features of included literatures

A total of 7 articles were incorporated in this study. Among these, the study of Nicol et al.^25^contained two sets of data, the study of Palladini et al.^26^contained three sets of data, and the other five studies contained one set of data each. Therefore, a total of 10 sets of data were included for further analysis. These seven studies, published from 2003 to 2020, involving 810 subjects in total (including 490 patients with CA involvement and 320 controls), were conducted in the Netherlands, Germany, France, Italy, among other countries. Moreover, the levels of NT-proBNP were all detected by immunoassay in the included studies. Among these, 4 studies focused on AL amyloidosis, 2 studies included TTR amyloidosis, while the other study incorporated both AL and TTR amyloidosis. Meanwhile, amyloidosis was confirmed by biopsy in 6 studies, not including Damy et al.,^21^who did not report a diagnostic strategy. Characteristics of these 7 studies, including the criteria of cardiac involvement and diagnostic thresholds were organized in Table 1 . Among the 7 included studies, 5^12 , 21 - 23 , 25^generated differential analysis on age of subjects, and 4 studies^12 , 21 - 23^compared the difference in gender between cases and controls. In these studies, Damy et al.^21^found significant differences in the gender and age of the study subjects (p < 0.05); samples included by Klaassen et al. were significantly different in age (p < 0.05); and other comparisons at p ≥ 0.05 were considered age- and/or gender-matched. Other three studies from Nicol^25^and Palladini et al.^24 , 26^did not compare differences in age and/or sex. We then used QUADAS 2 for the quality assessment of publications, and results showed a low risk of bias and a high quality of methodology of involved studies ( Supplemental Figure 1 ).

**Table 1:** – Characteristics of 7 included studies in this meta-analysis

Study	Area	Proof of amyloidosis	Type of amyloidosis	Criterion of cardiac involvement	N	Case/ Control			Cut-off, pg/ml	TP	FP	FN	TN
						n	Age, years	Male, n (%)					
CappelliF,^12^2014	Italy	Biopsy	AL	RVD	76	23/53	70.7±9.2/ 68.9±10.1	9 (39.1)/ 24 (45.3)	≥2977	20	8	3	45
Damy, T^21^2013	France	NR	TTR	LVD	36	26/10	65(56-74)/ 40 (33-56) *	20 (76.9)/ 3 (30.0) *	≥82	24	1	2	9
Klaassen, SHC^22^2017	The Netherlands	Biopsy	TTR	Structural myocardial wall abnormalities and/or conduction disturbances	77	39/38	59.3±10.9/ 46.1±13.0 *	25 (64.1)/ 18 (47.4)	≥125	36	13	3	25
Lehrke, S^23^2009	Germany	Biopsy	AL or TTR	Positive heart biopsy and/or LVH	34	25/9	55.5±11.0/ 59.8±7.8	10 (40.0)/ 6 (66.7)	≥1736.5	23	3	2	6
Nicol, M^25^2020	France	Biopsy	AL	CMR and endomyocardial biopsy	114	82/32	66 (58-73)/ 68 (60-76)	NR	≥850	75	8	7	24
					73	48/25	NR	NR	≥850	44	1	4	24
Palladini, G^24^2003	Italy	Biopsy	AL	Clinical symptoms of heart failure, LVH	152	90/62	61 (34-78) #	NR	≥152	84	6	6	56
Palladini, G^26^2012	Italy	Biopsy	AL	Left ventricular wall thickness >12 mm	109	62/47	62 (29-83) #	63 (58) #	≥332	62	5	0	42
					77	54/23	64 (35-85) #	34 (44) #	≥543	50	2	4	21
					62	41/21	65 (38-82) #	33 (53) #	≥2642	38	6	3	15

AL: amyloid light chain; TT: hereditary transthyretin-related; CMR: cardiac magnetic resonance imaging; RVD: Right ventricular dysfunction; LVD: Left ventricular dysfunction; LVH: left ventricular hypertrophy; NR: not reported; TP: true positive; FP: false positive; FN: false negative; TN: true negative. #, data of total sample. Statistical significances of all studies except Palladini et al.^24,26^were set at p < 0.05, and * indicates the statistical difference.

### Diagnostic value of NT-proBNP

A total of 7 articles (10 sets of population data) reported the results of NT-proBNP levels in the diagnosis of heart damage in patients with amyloidosis, and Spearman correlation analysis suggested a p = 1.00 as the result which indicated no significant threshold effect. A bivariate mixed effect model was then established to investigate the diagnostic value of NT-proBNP in heart damage based on different indicators, and Cochran’s and I^2^tests were applied for analysis on heterogeneity among studies. The results ( Figure 2 ) showed that the estimated sensitivity and specificity were 0.93 and 0.84, respectively. No significant heterogeneity was found in sensitivity (p = 0.67, I^2^= 0.0%); however, a significant heterogeneity was identified in specificity (p = 0.01, I^2^= 58.86%) across studies. In Figure 3 , the combined value of PLR was 5.77, with a significant heterogeneity between studies (p = 0.01, I^2^= 34.74%), while that for NLR was 0.80 with no significant heterogeneity (p = 0.79, I^2^= 0.0%). Figure 4A showed these data sets were significantly heterogeneous in DOR (p < 0.01, I^2^= 84.77%) with a combined estimate of 69.53. The AUC of SROC was 0.95, and these studies were not significantly distributed in a curvilinear shape ( Figure 4B ), suggesting a great diagnostic validity of NT-proBNP in heart damage.

**Figure 2:** – Forest plots of estimates of sensitivity (A) and specificity (B) in the diagnosis of NT-proBNP to heart damage from 10 sets of data.

**Figure 3:** – Forest plots of estimates of PLR (A) and NLR (B) in the diagnosis of NT-proBNP to heart damage from 10 sets of data.

**Figure 4:** – Diagnostic validity of NT-proBNP to heart damage. A) Forest plots of estimates of DOR from 10 sets of data. B) SROC curve showed the diagnostic validity of NT-proBNP in heart damage with an AUC of 0.95.

### Clinical utility of NT-proBNP

We further performed a Fagan’s nomogram to evaluate the clinical utility of NT-proBNP, as shown in Figure 5 , and the Fagan nomogram plot presented the pre-test probability, PLR, NLR, and post-test probability of NT-proBNP in the diagnosis of heart injury. The results suggested that the pre-test probability of patients with heart damage was 60.5%, while the post-test probability was 90% and 8% for positive and negative patients, respectively. That means after the diagnosis of NT-proBNP, the probability of developing heart damage in populations with NT-proBNP positive was 90%, while the possibility for NT-proBNP negative populations was only 8%.

**Figure 5:** – The clinical utility of NT-proBNP. The Fagan nomogram plot showed the pre-test probability, PLR, NLR, and post-test probability of NT-proBNP for the diagnosis of heart injury.

### Sensitivity analysis and publication bias test

A graph model was then conducted for sensitivity analysis. The results suggested a great residual-based goodness-of-fit of the model ( Figure 6A ), which basically conformed to the bivariate normality assumption ( Figure 6B ). This study also found that each independent study had no significant effect on the combined results of the model, and no outlier was identified ( Figure 6C-D ). Finally, a Deek’s funnel plot was created to test the publication bias, and results in Figure 7 suggested no significant publication bias with a p = 0.31 in the asymmetry test. These findings proposed stable and reliable combined results in this meta-analysis.

**Figure 6:** – Sensitivity analysis in a graph model. A-B) showed the goodness-of-fit and bivariate normality of the model. C) Influence analysis of independent study on the combined results. D) Outlier detection of independent study.

**Figure 7:** – Publication bias test. Deek’s funnel plot showed the publication bias in the asymmetry test.

## Discussion

Diagnosis of cardiac involvement for patients with amyloidosis is often delayed by the diversity of its clinical manifestations, thereby resulting in a poor prognosis.^5^It is reported that once AL amyloidosis presents symptoms of congestive heart failure, untreated patients have a median survival of less than 6 months.^2^Therefore, it is essential to develop a CA involvement-related biomarker to improve the efficiency of early diagnosis. NT-proBNP has been used as a potential biomarker to assess the severity of cardiac involvement in AL amyloidosis,^27^but the independent role in CA involvement and its diagnostic specificity have not been fully investigated. Hence, this meta-analysis was performed based on 7 articles, and evaluated the influence of NT-proBNP on the diagnosis of CA involvement. Our results suggested that NT-proBNP had significant diagnostic values for heart damage in patients with amyloidosis, with a sensitivity of 0.93, a specificity of 0.84, a PLR of 5.77, a NLR of 0.08 and a DOR of 69.53. The AUC of SROC curve was also close to 1 (0.95), thus demonstrating a great diagnostic validity of NT-proBNP.

It is reported that the local destruction of cardiomyocytes will lead to elevated levels of NT-proBNP, and the increasing NT-proBNP level could be considered as a predictor of cardiac involvement before the onset of heart failure.^7^Banypersad et al. also found a correlation between heart disease and NT-proBNP in 100 patients with AL amyloidosis scanned by nuclear magnetic resonance imaging.^28^Furthermore, 1-year mortality of 125 patients with AL amyloidosis can be predicted through the risk stratification analysis on NT-proBNP and cTn.^29^As for the potential regulation mechanism of NT-proBNP expression on amyloid in cardiomyocytes, Shi et al. proposed that light chain proteins isolated from amyloid cardiomyopathy tissues may induce p38 mitogen activated protein kinase (MAPK) signal, thereby contributing to the oxidative stress and death of cardiomyocytes.^30^Moreover, for AL amyloidosis, MAPK signal could mediate BNP transcription, and their interaction may support the cardiotoxic effect of light chain proteins.^31^Combined with the above findings, it can be speculated that the expression of NT-proBNP could be directly regulated by the MAPK signal transduction pathway induced by light chain proteins in cardiomyocytes, and the increased expression level of NT-proBNP can predict the attack of heart failure.

A variety of studies have focused on the influence of NT-proBNP on cardiac involvement, including heart failure, cardiomyopathy, and myocardial infarction. A related meta-analysis reported that the combined sensitivity and specificity of NT-proBNP level in differentiating heart failure associated effusion was 94%, with a PLR of 15.2 and a NLR of 0.06.^32^The increasing level of NT-proBNP also shows a strong ability to predict the prognosis of cardiomyopathy.^33^Additionally, by comparing to revised cardiac risk index, the high-sensitivity biomarker NT-proBNP can improve the prediction of myocardial infarction after major non-cardiac surgery.^34^These findings supported our conclusions, but Januzzzi et al. further proposed that the level of NT-proBNP was correlated with the severity of heart failure symptoms, and the sensitivity and specificity of heart failure varied between different age groups.^35^In this study, no direct relationship was observed between age and NT-proBNP levels, and we were unable to confirm the importance of age in the CA involvement diagnosed by NT-proBNP. Additionally, studies also found that female subjects have higher levels of NT-proBNP than age-matched male subjects.^36^Therefore, a stratified analysis will be conducted at a future moment to explore the differences of NT-proBNP markers based on analytical performance, so as to provide more accurate diagnostic information for patients with CA involvement at different clinical stratification.

The virtue of this study included that the incorporated study was highly qualified in methodology, and the confounding bias was minimal. Furthermore, there was no significant publication bias in this study, and the influence analysis also suggested that the combined results were not affected by each independent study. More importantly, the combined results of all indicators were relatively consistent, suggesting that NT-proBNP had a high application value in the diagnosis of heart damage in patients with amyloidosis, and the results were stable and reliable. Although our results suggested high sensitivity and specificity of NT-proBNP in the diagnosis of CA involvement, the significant heterogeneity in specificity, PLR and DOR between included studies was one of the limitations. Meanwhile, there were also differences in diagnostic criteria, types of amyloidosis and criteria for determining cardiac damage among the subjects. However, due to limited simple size of included literatures, it is difficult to explore the source of heterogeneity through quantitative methods, such as meta-regression. Secondly, all included studies were carried out based on the population of Europe with a poor generalization of results. High-quality studies are still needed in Asia, Africa, and other regions to validate the performance of results.

## Conclusion

In conclusion, this study suggested that NT-proBNP played a positive role in the early diagnosis of cardiac involvement in patients with amyloidosis. Large-scaled studies in other regions and races are needed to verify the extrapolation of the results.

## * Supplemental Materials

See the Supplemental Figure, please, click here



See the Supplemental Tables, please, click here.
